# Molecular characterization of *Trichinella spiralis* aminopeptidase and its potential as a novel vaccine candidate antigen against trichinellosis in BALB/c mice

**DOI:** 10.1186/1756-3305-6-246

**Published:** 2013-08-23

**Authors:** Ya Lan Zhang, Zhong Quan Wang, Ling Ge Li, Jing Cui

**Affiliations:** 1 Department of Parasitology, Medical College, Zhengzhou University, 40 Daxue Road, Zhengzhou 450052, P. R. China

**Keywords:** *Trichinella spiralis*, Aminopeptidase, Immune protection, Trichinellosis

## Abstract

**Background:**

*Trichinella spiralis* is an intracellular parasite that can cause a serious threat to human health by causing trichinellosis. The aminopeptidase (AP) was found in the proteins produced by *T. spiralis* infective larvae after *in vitro* co-culture with intestinal epithelial cells, but its characteristics and function are unknown. The purpose of this study was to identify the *T. spiralis* aminopeptidase (TsAP) and to investigate its potential as a vaccine candidate antigen against *T. spiralis* infection.

**Methods:**

*T. spiralis* aminopeptidase (TsAP) gene encoding a 54.7 kDa protein was cloned and expressed in *Escherichia coli*, and purified recombinant TsAP protein was used to immunize BALB/c mice. The antibodies obtained were used to determine where TsAP was localized in the parasite. Transcription and expression of TsAP in different developmental stages of *T. spiralis* were observed by RT-PCR and Immunofluorescence test (IFT). The immune protection of recombinant TsAP protein against *T. spiralis* infection in BALB/c mice was evaluated.

**Results:**

Anti-TsAP antibodies recognized the native protein migrating at 54.7 kDa by Western blotting of the crude antigens from muscle larvae. Transcription and expression of TsAP gene was observed in different developmental stages (adult worms, newborn larvae, pre-encapsulated larvae and muscle larvae). TsAP appears to be a cytoplasmic protein located primarily at the cuticle and internal organs of this parasite. After a challenge infection with *T. spiralis* infective larvae, mice immunized with the recombinant TsAP protein displayed a 38.1% reduction in adult worm burden and 59.1% reduction in muscle larval burden.

**Conclusions:**

In this study, *T. spiralis* aminopeptidase (TsAP) was first characterized and will help reveal its potential biological functions. TsAP is a novel potential vaccine candidate antigen that merits further investigation.

## Background

*Trichinella spiralis* is an intracellular parasitic nematode of mammalian skeletal muscles. The infective larvae invade the epithelium of the small intestine, where they mature to the adult stage, mate, and produce newborn larvae, which enter the blood and migrate to striated muscle where they grow and mature to the infective stage, thus completing the life cycle [1,2]. It is well known that the invasion of host intestinal epithelial cells (IECs) by the infective larvae is the first step during *T. spiralis* infection. Since the larvae do not possess oral appendices or a spike [3], it is implied that the larval invasion of IECs may not simply be a result of mechanical penetration. Some studies have shown that the invasion of intestinal epithelia by *T. spiralis* can be inhibited by antibodies against the excretory-secretory (ES) antigens, suggesting that these ES antigens may play an important role in the invasion and developmental process of *Trichinella* larvae [4-7]. However, the mechanisms by which *T. spiralis* infective larvae recognize, invade, and migrate within the intestinal epithelia are unknown.

Previous studies have shown that proteolytic enzymes are present in the ES products of *T. spiralis* muscle larvae (ML) [8]. Several proteases (such as serine and cysteine proteases) have been identified to possess collagenolytic and elastolytic activities and were inhibited by IgG molecules isolated from mice infected with *T. spiralis*, an observation of relevance to understanding the host-parasite interaction [9,10]. Our previous studies showed that when the *T. spiralis* infective larvae were inoculated onto monolayers of IECs, they invade the IECs and produced several proteins, and some of these proteins entered the IECs [11,12]. Out of the proteins produced by the infective larvae after co-culture with IECs, *T. spiralis* aminopeptidase (TsAP, GenBank accession No. EFV57052) was identified by shotgun LC-MS/MS. TsAP is a kind of proteolytic enzyme, it can catalyze the amino acid release from the N-terminus of a polypeptide chain and plays an important role in the degradation of some bioactive peptides [13]. TsAP might be related with the larval invasion of IECs and mediate or facilitate the entry into cells.

In the present study, the TsAP gene encoding a 54.7 kDa protein from *T. spiralis* muscle larvae was cloned and identified. The expression, immunolocalization of TsAP and the immune protection conferred by the recombinant TsAP protein was also investigated.

## Methods

### Parasites and experimental animals

The isolate (ISS534) of *T. spiralis* used in this study was obtained from domestic pigs in Nanyang, Henan Province, China. The *Trichinella* isolate was maintained by serial passage in Kunming mice every 6–8 months. Specific pathogen-free (SPF) male BALB/c mice aged 5 weeks were purchased from the Experimental Animal Center of Henan Province and used for the immunological studies and challenge infection.

### Collection of worms and preparation of crude and ES antigens

*T. spiralis* muscle larvae (ML) from infected mice at 42 days post-infection (dpi) were recovered by digestion of carcasses with 0.33% pepsin (1:31000; Sigma) and 1% HCl [14]. The pre-encapsulated larvae (PEL) at 19 dpi were isolated using Baermann’s method [15]. Adult worms (AW) were isolated from the small intestines of infected mice at 3 dpi [16]. The newborn larvae (NBL) were collected from female adult worms and cultured in RPMI-1640 medium containing 10% fetal bovine serum (FBS; Gibco) in 5% CO_2_ at 37°C for 24 h [17].

The crude and ES antigens of the ML were prepared as previously described [11]. In brief, after washing thoroughly in sterile saline, the larvae were again washed four times in serum-free RPMI-1640 medium supplemented with 100 U penicillin/ml and 100 μg streptomycin/ml. The larvae were incubated in the same medium at a concentration of 5000 worms/ml for 18 h at 37°C in 5% CO_2_. After incubation, the media containing the ES products were filtered through a 0.2 μm membrane into a 50 ml conical tube, then centrifuged at 4°C, 15,000 × g for 30 min. The supernatant was dialyzed against deionized water at 4°C for 2 days, and then concentrated by a vacuum concentration and freeze drying (Heto Mxi-Dry-Lyo, Denmark), respectively. The protein concentration of ES antigens (1.26 mg/ml) was determined by the method described by Bradford [18].

### Cloning, expression, and identification of TsAP

Total RNA was extracted from the ML using Trizol (Invitrogen). The first-strand synthesis of cDNA was accomplished using AMV reverse transcriptase (Promega, USA) and oligo (dT) primers at 42°C for 1 h according to the manufacturer’s instructions. The TsAP gene was amplified by PCR, and specific primers carrying *Bam*HI and XhoI restriction enzyme sites (Forward, 5′-ATAGGATCCATGAGCCGCAAAGGATTGATG-3′; Reverse, 5′-CCGCTC GAGTCAACTAGATTTTGCCAAAAG-3′) were used. The cycling protocol was as follows: 30 cycles of 94°C for 45 s, 58°C for 45 s and 72°C for 1.5 min. The purified PCR products were cloned into the expression vector pGEX-6p-1 (Novagen, USA) using the *Bam*HI and XhoI sites. The recombinant plasmid was then transformed into *Escherichia coli* BL21 (Novagen, USA). The expression of the recombinant protein was induced with 0.5 mM IPTG at 37°C for 4 h, with the formation of insoluble inclusion bodies. The inclusion bodies were recovered from the bacterial lysates by centrifugation at 12,000 *g* for 10 min and dissolved in 8 M urea. After denaturing in urea, gradient dialysis was used for protein renaturation. Then the refolded proteins were purified by Glutathione Sefinose resin (Bio Basic Inc, Canada). The purified recombinant proteins were analyzed by sodium dodecyl sulfate polyacrylamide gel electrophoresis (SDS–PAGE) using a 5% acrylamide stacking gel and 12% acrylamide separating gel (83 × 73 × 1.0 mm) with a Mini-PROTEAN 3 Cell electrophoresis unit (BioRad, USA) at 120 V for 2.5 h [11]. After electrophoresis, the gel was stained with 0.25% Coomassie brilliant blue R-250 for 4 h and then destained (10% acetic acid and 5% ethanol). Another gel was prepared in the same way and used for the immunoblotting described below.

### Generation of antibodies to recombinant TsAP protein, ES and surface antigens

A total of 20 male BALB/c mice were divided into two groups of 10 mice each. Pre-immune sera were collected by tail bleeding 2 days prior to the first immunization. The BALB/c mice were subcutaneously immunized with 20 μg of recombinant TsAP proteins or ES antigens, followed by three boosts with the same amount of protein at 10-day intervals. Seven days after the last boost, the mice were bled, and the sera were collected.

### Antibody determination

The specific IgG antibodies to TsAP in serum of immunized mice were determined by ELISA using corresponding recombinant, ES or crude antigens. The procedure was performed as previously described [19]. Briefly, microtiter plates (Nunc) were coated with recombinant TsAP proteins, ES or crude antigens (2.5 μg/ml) in coating buffer overnight at 4°C, and blocked with 200 μl of PBS-0.1% Tween 20 (PBST) containing 5% skimmed milk. Then, 100 μl of immune serum samples with 1:100 dilution in PBS were added to each well and incubated at 37°C for 1 h. HRP-conjugated goat anti-mouse IgG antibodies (1:5000; Southern Biotechnology, USA) were added and incubated for 1 h at 37°C. The plates were developed with o-phenylenediamine dihydrochloride substrate (OPD; Sigma), and the absorbance was measured at 490 nm.

### Western blot analysis

Samples including crude antigens, ES antigens and the recombinant proteins were separated by SDS–PAGE and then transferred onto nitrocellulose membranes (Millipore, USA) using a trans-blot SD transfer cell (Bio-Rad, USA) [20]. The membranes were cut into strips, blocked with 5% skimmed milk in Tris-Buffered Saline with Tween-20 (TBST) at 37°C for 1 h, and incubated at 37°C for 1 h with 1:100 dilution of different mouse sera (anti-TsAP serum, serum from mice infected *T. spiralis* at 30 dpi and normal mouse serum). After washing, the strips were incubated at 37°C for 1 h with HRP-conjugated goat anti-mouse IgG (1:5000 dilution; Southern Biotechnology, USA), and finally with 3, 3’-diaminobenzidine tetrahydrochloride (DAB; Sigma).

### RT-PCR analysis of TsAP gene transcription

To observe the transcription of the TsAP gene at different developmental stages of *T. spiralis*, total RNA was extracted from the ML at 42 dpi, PEL at 18 dpi, AW at 3 dpi and NBL of *T. spiralis*. RT-PCR was performed as previously described [21]. The housekeeping gene GAPDH (glyceraldehyde-3-phosphate dehydrogenase, GenBank accession No. AF452239) of *Trichinella* was used as a constitutively expressed standard gene. The primers were designed as follows: forward, 5′-TTAATGTCGTGGCTGTGAAT-3′, and reverse, 5′-CCAGTAGAAG CAGGGATGAT-3′.

### Immunofluorescence test (IFT)

IFT was used to observe the expression of TsAP at different developmental stages and its immunolocalization in the parasite. The skeletal muscles from mice infected with *T. spiralis* were collected at 19 and 42 dpi, respectively, and were fixed in 4% paraformaldehyde and embedded in paraffin. Microtome-cut 4-μm sections were placed on slides, deparaffinized in xylene and rehydrated. The whole parasites and tissue sections were blocked with 5% normal goat serum in PBS and then incubated in a moist chamber at 37°C for 1 h with a 1:10 dilution of immune and normal sera. After washing three times in PBS, the whole parasites and sections were incubated with a 1:50 dilution of FITC-labeled goat anti-mouse IgG (Santa Cruz, USA), washed five times in PBS, and examined under a fluorescent microscope (Olympus, Japan) [11].

### Immune protection against challenge infection

To determine the immune protection of the recombinant TsAP protein, a total of 48 male BALB/c mice were divided into three groups (immune group, adjuvant control group and PBS control group) of 16 mice each. Pre-immune sera were collected by tail bleeding 2 days prior to the first immunization. The immune groups of BALB/c mice were subcutaneously immunized with 20 μg of purified recombinant TsAP protein. The vaccines were prepared at a 1:1 ratio by mixing each antigen in Freund’s complete adjuvant for the first immunization or Freund’s incomplete adjuvant for the subsequent immunizations (three boosts at 10 day- intervals) and administered intraperitoneally and intradermally at multiple sites of the abdomen. The control group received PBS with the corresponding adjuvant or PBS only. Ten days after the last immunization, all the three groups of mice were orally challenged with 300 *T. spiralis* muscle larvae. Eight mice from each group were euthanized 3 days after challenge and the numbers of intestinal adult worms were counted [22]. The muscle larvae were examined from another 8 mice from each group 42 days after challenge by artificial digestion of all the whole carcass of each infected mouse [14]. The protective immunity was calculated as the worm reduction rate of recovered adults and larvae per gram (LPG) muscle from the immunized groups versus those from the control groups [19].

### Statistical analysis

All of the statistical analyses of the data were performed using SPSS for Windows, version 17.0 (SPSS Inc., Chicago, IL). The AW and ML recovery data were expressed as the mean value ± standard deviation (SD), and the differences among the groups were analyzed using the one-way ANOVA method. When a pairwise comparison was carried out, the Fisher’s least significant test was used. The statistical significance was defined as *P* < 0.05.

## Results

### Molecular cloning and expression of a cDNA encoding TsAP

**Figure 1:**
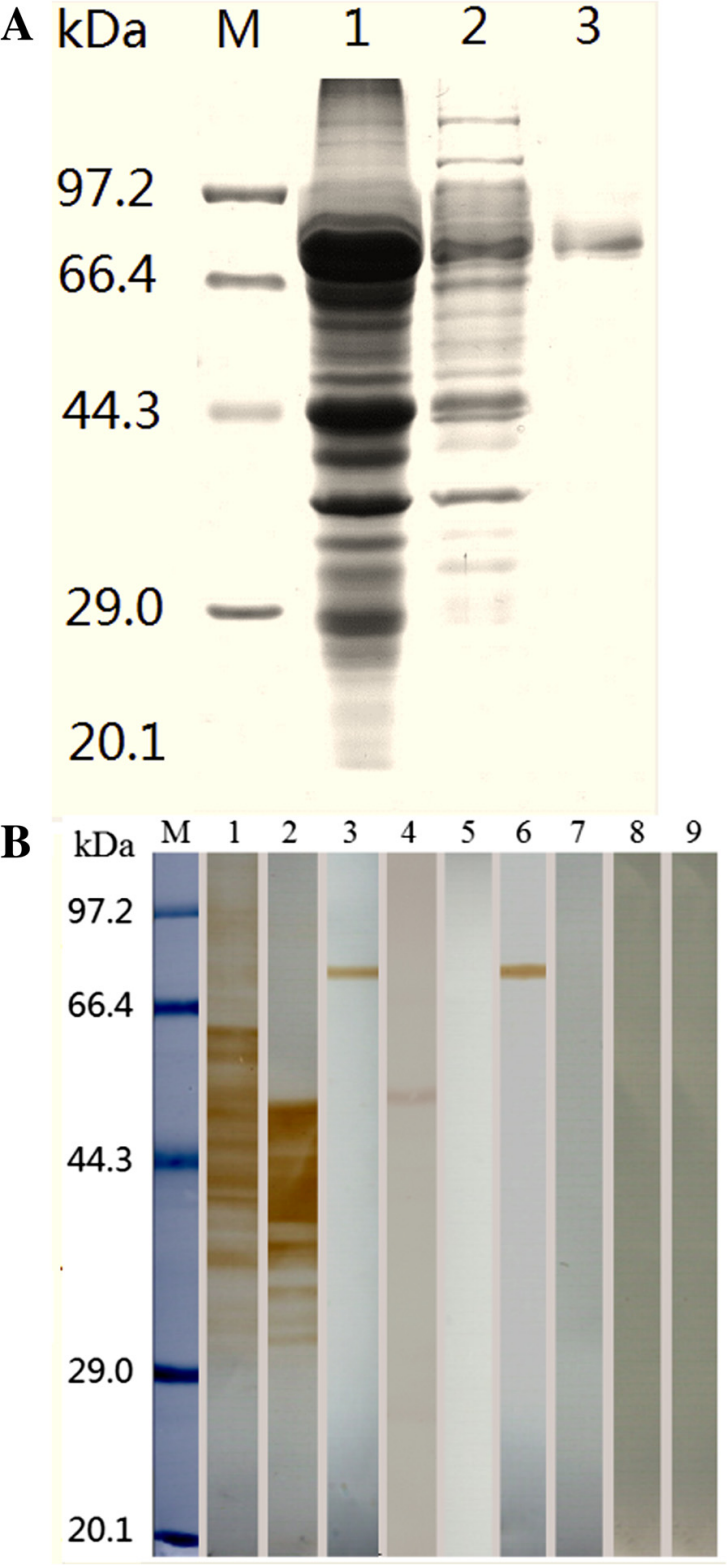
**Identification of recombinant TsAP protein. (A)** SDS-PAGE analysis of recombinant TsAP protein. M: protein molecular weight marker; 1: the lysis of the induced recombinant bacteria after ultrasonication; 2: TsAP inclusion body dissolved in 8 M urea; 3: recombinant TsAP protein purified by GST Sefinose Resin. **(B)** Western-blot analysis of recombinant TsAP protein antigenicity. The *T. spiralis* crude antigens (lane 1), ES antigens (lane 2), and recombinant TsAP protein (lane 3) were recognized by sera of mice infected with *T. spiralis* at 30 dpi*.* The native TsAP protein in crude antigens (lane 4) and recombinant TsAP protein (lane 6) were recognized by anti-TsAP serum, but the ES antigens (lane 5) were not recognized by anti-TsAP serum. The *T. spiralis* crude antigens (lane 7), ES antigens (lane 8), and recombinant TsAP protein (lane 9) were not recognized by sera of normal mice.

The cDNA sequence of TsAP gene is 1515 bp. The Blast analysis showed that the TsAP gene cloned in this study was 97% identical to the sequence of TsAP gene in GenBank. The full coding sequence of TsAP gene was cloned into the prokaryotic expression plasmid pGEX-6p-1. After induction with 1 mM IPTG, BL21 bacteria harboring pGEX-6p-1-TsAP expressed a 80.7 kDa fusion protein. Using SDS-PAGE, the molecular size of the recombinant protein was consistent with the predicted combined size of the polypeptide encoded by the cDNA clone (54.7 kDa) and N-terminal GST tag from the vector (26 kDa) (Figure 1A).

### Western blot and ELISA analysis of the recombinant TsAP protein

**Figure 2:**
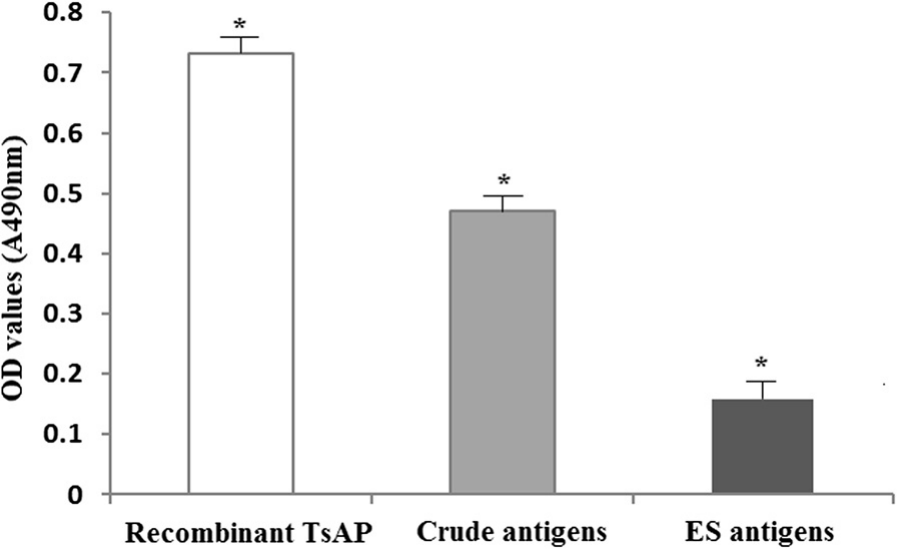
**The specific IgG antibody levels of mice immunized with recombinant TsAP protein assayed by ELISA using different antigens.** The optical density (OD) values shown for each group are the mean ± standard deviation (SD) of antibody levels (n = 13). Asterisks (*) indicate statistically significant differences (*P* < 0.01) in OD values of one kind of antigens compared to other two kinds of antigens.

The Western blot analysis showed that the recombinant TsAP protein was recognized by an anti-TsAP serum and serum of mice infected *T. spiralis*. The 54.7 kDa protein component of the crude antigens of *T. spiralis* ML were recognized by the anti-TsAP serum (Figure 1B). The results of the ELISA showed that anti-TsAP serum recognized the recombinant TsAP protein and crude antigens, but did not recognize the ES antigens (Figure 2). The results indicated that TsAP is one component of the crude antigens but not from ES antigens.

### RT-PCR analysis

**Figure 3:**
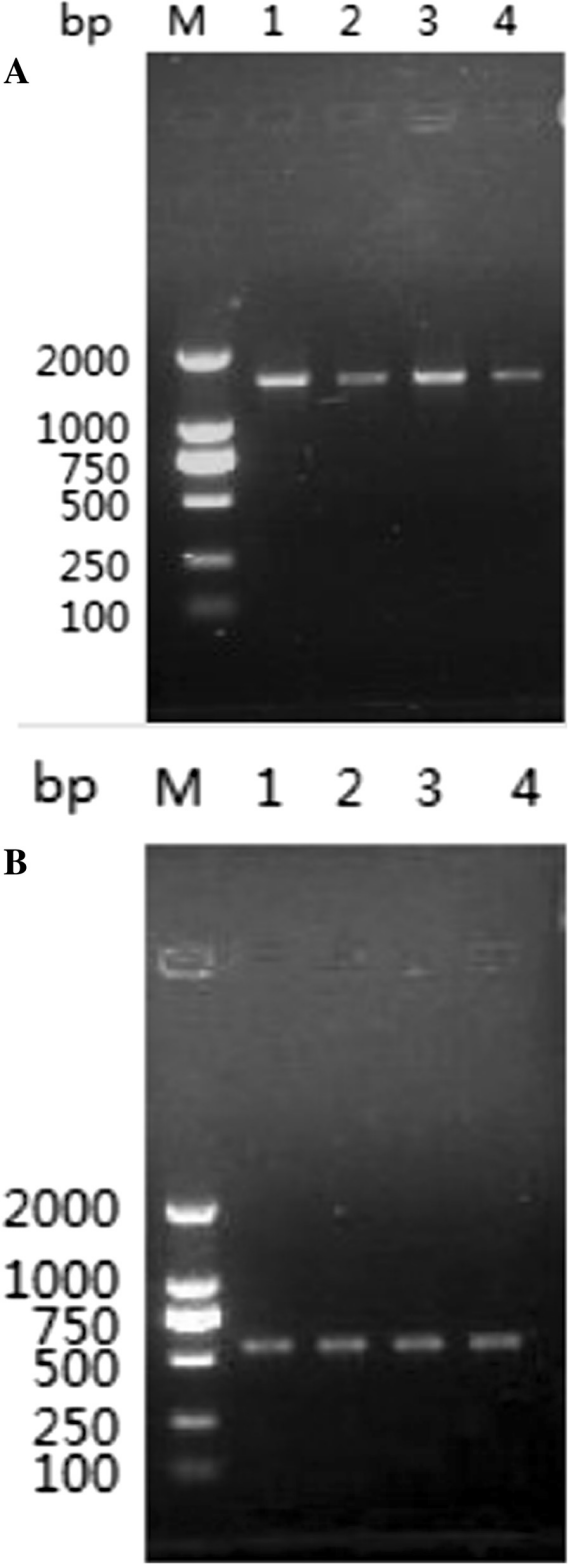
**RT-PCR analysis of TsAP gene transcript at different stages of *T. spiralis*.** RT-PCR detection of mRNA transcription for the TsAP gene **(A)** and GAPDH gene **(B)** at different developmental stages of *T. spiralis*. M: DNA marker; Lane 1: AW at 3 dpi; Lane 2: ML at 42 dpi; Lane 3: PEL at 19 dpi; Lanes 4: NBL.

The transcript of the TsAP gene at different developmental stages of *T.spiralis* was determined using RT-PCR with the transcript of housekeeping gene GAPDH as a control. The mRNA transcript (1515 bp) for the TsAP gene was detected at all of the developmental stages (e.g., AW at 3 dpi, NBL, PEL at 19 dpi and ML at 42 dpi) (Figure 3A). Furthermore, the primers for a standard gene (GAPDH) generated the expected size (570 bp) band in all of the samples (Figure 3B).

### Expression of TsAP at different developmental stages and immunolocalization

**Figure 4:**
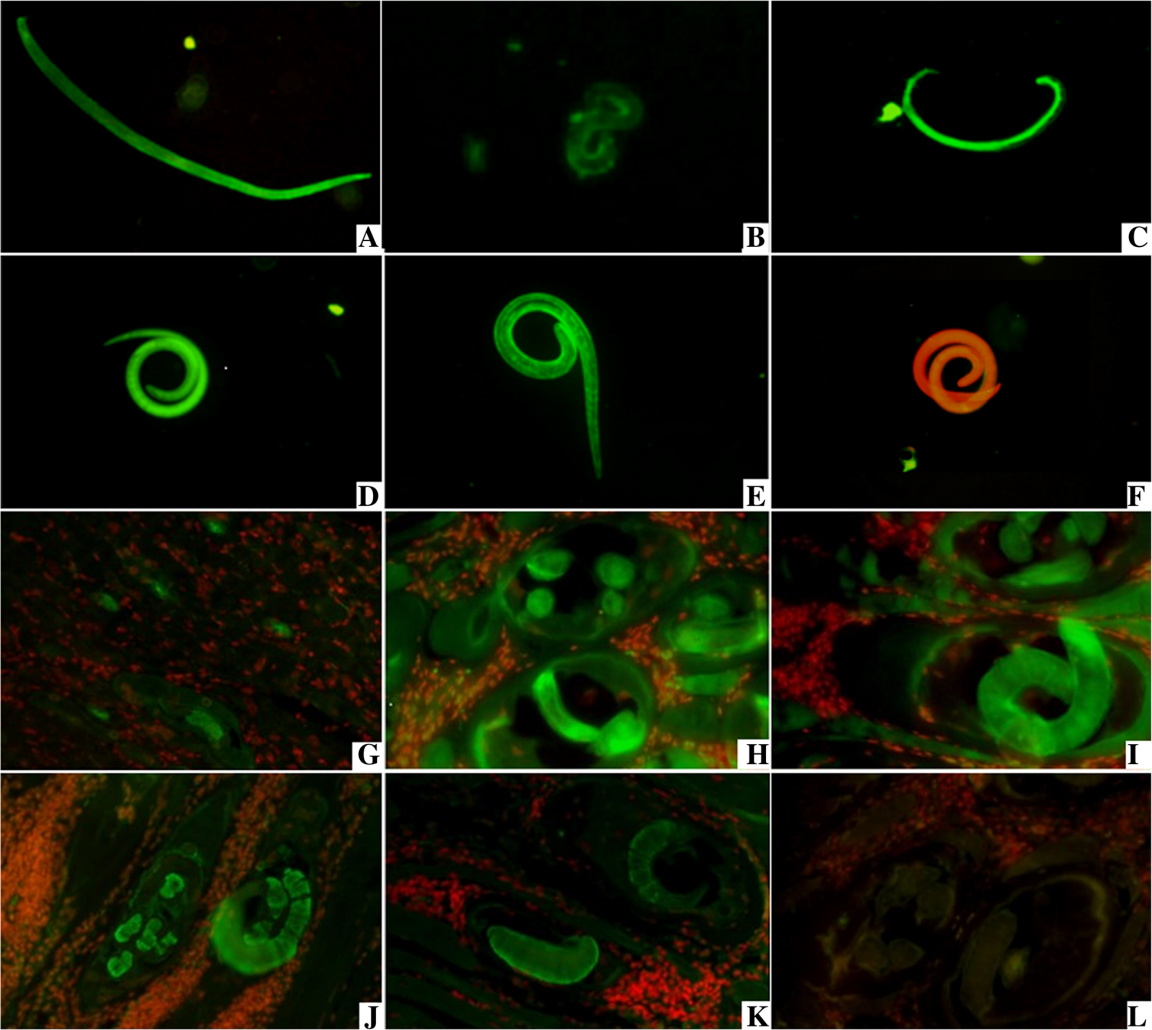
**Expression of TsAP at different developmental stages and immunolocalization in *T. spiralis.*** A–H: The results of IFT with whole parasite of *T. spiralis* different stages reacted with anti-TsAP serum. The notable immunostaining is found in the body of AW at 3 dpi **(A)**, NBL **(B)**, PEL at 19 dpi **(C)** and ML at 42 dpi **(D)**. The ML at 42 dpi reacted with serum of mice infected *T. spiralis* at 30 dpi **(E)** as a positive control; ML at 42 dpi did not show recognition by normal serum **(F)** as a negative control. G–L: The results of IFT with the sections of skeletal muscles of infected mice reacted with anti-TsAP serum. The immunostaining is seen at the cuticle and internal organs of PEL at 19 dpi **(G)** and ML at 42 dpi **(H and I)**. The ML at 42 dpi reacted with anti-ES serum **(J)** and serum of mice infected *T. spiralis* at 30 dpi **(K)** as a positive control, ML at 42 dpi did not did not show recognition by normal mouse serum **(L)** as a negative control.

The results of IFT with the whole parasite showed that the intense staining using anti-TsAP serum was found in the body of all the different developmental stages of *T. spiralis* (e.g., AW at 3 dpi, NBL, PEL at 19 dpi and ML at 42 dpi) (Figure 4). When the sections of skeletal muscle tissues of infected mice were incubated with the anti-TsAP serum, positive staining was found at the cuticle and internal organs of PEL at 19 dpi and ML at 42 dpi (Figure 4).

### Immune protection of recombinant TsAP against challenge infection

**Figure 5:**
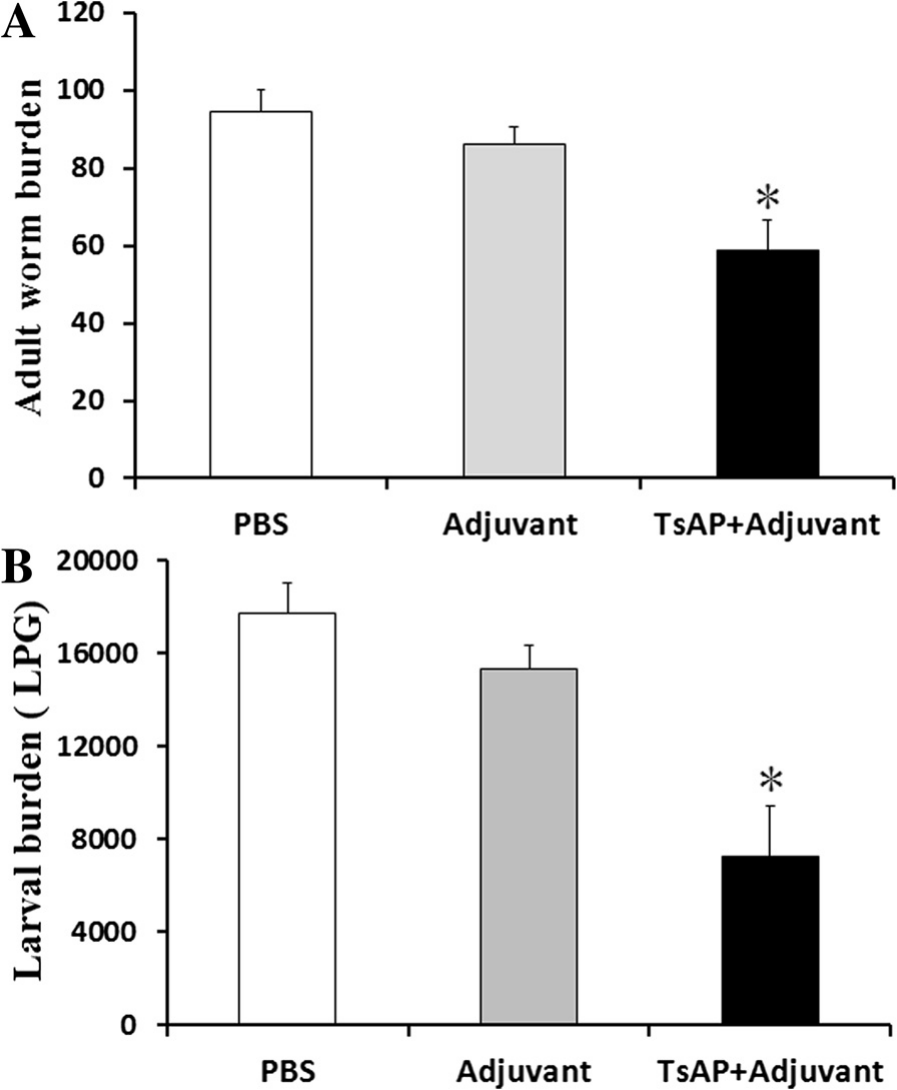
**The number of adult worms (A) and larvae per gram (lpg) muscles (B) recovered from vaccinated mice after a challenge infection with 300** ***T. spiralis *larvae.** Results are presented as the arithmetic mean ± standard deviation (SD) of eight mice in each group. Asterisks (*) indicate statistically significant differences (*P* < 0.01) in worm recovery of the immunized group compared to both control groups.

Protective immunity against *T. spiralis* infection induced by the recombinant TsAP protein was observed in immunized BALB/c mice. After the challenge infection with *T. spiralis* infective larvae, the mice immunized with the recombinant TsAP protein displayed a 38.1% reduction in their adult worm burden (Figure 5A) and 59.1% reduction in the muscle larval burden (Figure 5B) compared with the groups immunized with adjuvant or PBS alone; these differences were statistically significant (*F*_adults_ =47.38, *F*_larvae_ =64.23, *P* < 0.05).

## Discussion

In the present study, the TsAP gene encoding a 54.7 kDa protein from *T. spiralis* was successfully cloned and expressed in an *E. coli* expression system, and the resulting recombinant protein and immune serum were used to define some characteristics of the native 54.7 kDa protein of *T. spiralis*. The expression at high levels of a foreign protein in *E. coli* results in the formation of inclusion bodies composed of insoluble inactive aggregates of the expressed protein; however, after purification, such recombinant protein has a good immunogenicity in mice and can be used as an immunogen to produce antibodies [21]. In this study, the BALB/c mice immunized directly with the purified recombinant TsAP protein produced strong specific antibodies against the recombinant TsAP protein. Our Western blot analysis showed that the anti-TsAP serum obviously recognized the 54.7 kDa band in the crude antigens of *T. spiralis* muscle larvae. The results of the ELISA also indicated that the crude antigens strongly reacted with anti-TsAP serum, but the ES antigens were not recognized by anti-TsAP serum. Our results demonstrated that the TsAP protein might be one of components of the somatic antigens of *T. spiralis* ML.

The characteristics of TsAP were identified at the gene transcription and protein expression levels using RT-PCR and IFT. As shown in Figure 3, the results of RT-PCR showed that the TsAP mRNA is transcribed during all the different developmental stages of *T. spiralis* (AW, ML, NBL and PEL). The IFT revealed positive stainings were widely distributed in the body of the whole parasites (AW, ML, NBL and PEL) or sections of infected muscles incubated with the anti-TsAP serum. Aminopeptidases are exopeptidases that catalyze the sequential removal of amino acids from the N termini of peptides and play a major role in regulating the balance between catabolism and anabolism in all living cells [13]. The aminopeptidases belong to Peptidase M17 family, which can also be classified into different aminopeptidases according to its major substrate. Leucine aminopeptidases (LAP) are the representative group of aminopeptidases [13]. They have been identified, purified and characterized in many helminth and protozoal parasites [23,24] and shown to play important roles such as molting, surface membrane remodeling, egg hatching and digestion for the survival of parasites within host [25-27]. The tissue localization and functional analysis of LAP in *Setaria cervi* and several other nematodes also suggested that this enzyme is involved in parasite feeding, final digestion of the partially hydrolysed peptide fragments within gastrodermal cells, cuticle remodeling, egg hatching and embryogenesis [28,29]. However, to the best of our knowledge, there has been no report of the characterization and functional analysis of TsAP. The protein sequences of TsAP were compared with the protein database by the search protein database (http://blast.ncbi.nlm.nih.gov/). The results showed that the TsAP protein is one of the number of cytoplasmic aminopeptidase families, distributed in the cytoplasm, and might play an important role in the larval invasion and development of *T. spiralis*.

Our results showed that TsAP gene was transcribed and expressed during all the different developmental stages of *T. spiralis*, suggesting that the TsAP is an indispensable protein and plays an important role in the life cycle of *T. spiralis*. Furthermore, after the challenge infection with *T. spiralis* infective larvae, the mice immunized with the recombinant TsAP protein displayed a 38.1% reduction in adult worm burden and a 59% reduction in muscle larval burden. The ML reduction observed in this study is higher than previously reported [19,21,30,31], which might be related to the different expression vectors used. In the present study, the recombinant TsAP protein carried GST-tag, which might have antigenicity and increase the immune protection. Additionally, attenuated *Salmonella* strains are especially attractive live vectors because they elicit mucosal immunity, which is known to be important for the control of *T. spiralis* infection during the intestinal stage and can be administered by oral or intranasal routes. The mice immunized by intranasal route with the recombinant *Salmonella* carrying a *T. spiralis* gp43 antigen-derived 30-mer epitope elicited a protective immune response against challenge infection, reducing by 61.83% the adult burden at 8 dpi [32]. Other study showed that the vaccination with only LAP purified from *Fasciola hepatica* adults induced high levels of protection (89%) against fascioliasis in sheep [33]. The results showed that the recombinant TsAP protein induced a partial protective immunity in mice and could be considered as a novel potential vaccine candidate antigen against *T. spiralis* infection.

## Conclusions

For the first time, we have presented the molecular characterization of the aminopeptidase from *T. spiralis.* The TsAP gene was transcribed and expressed during all the different developmental stages of *T. spiralis*, suggesting that the TsAP is an indispensable protein and plays an important role in the life cycle of *T. spiralis*. TsAP appears to be a cytoplasm protein located primarily at the cuticle and internal organs of this parasite. The results of this study show that mice vaccinated with the recombinant TsAP protein could induce partial protective immunity against *T. spiralis* infection, indicating TsAP might be a novel potential vaccine candidate antigen.

### Ethics statement

All of the animal experiments reported herein were approved by The Life Science Ethics Committee of Zhengzhou University.

## Competing interests

The authors declare that they have no competing interests.

## Authors’ contributions

ZQW and JC conceived and designed the experiments. YLZ, LGL, ZQW, and JC performed the experiments. YLZ, ZQW, and JC, analyzed the data and wrote the manuscript. All authors read and approved the final version of the manuscript.
